# An Integrated Ontology Resource to Explore and Study Host-Virus Relationships

**DOI:** 10.1371/journal.pone.0108075

**Published:** 2014-09-18

**Authors:** Patrick Masson, Chantal Hulo, Edouard de Castro, Rebecca Foulger, Sylvain Poux, Alan Bridge, Jane Lomax, Lydie Bougueleret, Ioannis Xenarios, Philippe Le Mercier

**Affiliations:** 1 SIB Swiss Institute of Bioinformatics, CMU, University of Geneva Medical School, Geneva, Switzerland; 2 European Molecular Biology Laboratory, European Bioinformatics Institute (EMBL-EBI), Wellcome Trust Genome Campus, Hinxton, United Kingdom; CSIRO, Australia

## Abstract

Our growing knowledge of viruses reveals how these pathogens manage to evade innate host defenses. A global scheme emerges in which many viruses usurp key cellular defense mechanisms and often inhibit the same components of antiviral signaling. To accurately describe these processes, we have generated a comprehensive dictionary for eukaryotic host-virus interactions. This controlled vocabulary has been detailed in 57 ViralZone resource web pages which contain a global description of all molecular processes. In order to annotate viral gene products with this vocabulary, an ontology has been built in a hierarchy of UniProt Knowledgebase (UniProtKB) keyword terms and corresponding Gene Ontology (GO) terms have been developed in parallel. The results are 65 UniProtKB keywords related to 57 GO terms, which have been used in 14,390 manual annotations; 908,723 automatic annotations and propagated to an estimation of 922,941 GO annotations. ViralZone pages, UniProtKB keywords and GO terms provide complementary tools to users, and the three resources have been linked to each other through host-virus vocabulary.

## Introduction

Viruses are genetic entities that infect all kinds of organism. Their impact on living beings is huge, affecting human health, agricultural and other economic activity, global ecology, and even evolution. Exploration of the viral world is still very much in its infancy, with new species being discovered all the time - 138 new species have been officially recognized between 2011 and 2012, raising the total number of referenced viral species to 2618 (http://ictvonline.org/virusTaxInfo.asp). Advances in high-throughput DNA sequencing have resulted in an explosion in the number of virus genome sequences deposited in public databases over the past decade [1]. Detailed human- and machine-readable annotation of viral genome sequences – including known geographical sites of isolation, host specificity and interactions, functions and roles of individual viral proteins, and sequence variants – are essential to extract the maximum value from this data deluge, but the majority of available contextual data is published in papers or reviews in textual form that can only be interpreted by humans. Expert curation consists in associating viral sequences with experimental knowledge expressed in the form of human-readable text, ontologies and controlled vocabularies, which are searchable and even amenable to interpretation by machines. This requires human experts with deep knowledge of the underlying biology and a clear understanding of how to express and encode that knowledge in a consistent manner. Curators also perform an editorial function, acting to highlight (and where possible resolve) conflicting reports - one of the major added values of manual annotation.

ViralZone is a database that links virus sequence data with knowledge of virus molecular biology curated from peer-reviewed literature using human-readable text and controlled vocabularies. This web resource was created in 2009 and has been continually developed since that time by the viral curation team of the SwissProt group. The core of ViralZone is the virus fact sheet that describes the virion, genome, replication cycle and host data for each of the known virus genera and families. Virus molecular biology pages describe viral processes such as viral entry by endocytosis and viral genome replication in detail, with graphical illustrations that provide a global view of each process and a listing of all known viruses which conform to the particular schema. ViralZone pages are linked to protein sequence records of the UniProt Knowledgebase (mostly reference proteomes). This provides facile access to expert-curated information on individual protein sequences and their functions in textual form and as controlled vocabularies and ontologies. Ontologies consist of hierarchized controlled vocabulary in computer-friendly format. They provide a frame for global annotation, and facilitate analysis of biological data. In the era of metagenomics and large-scale studies, ontologies are an extremely potent tool to link knowledge with gene products and help identifying common patterns. UniProtKB keywords constitute an ontology with a hierarchical structure designed to summarize the content of an entry and facilitate the search of proteins of interest. They are classified in 10 categories: Biological process, Cellular component, Coding sequence diversity, Developmental stage, Disease, Domain, Ligand, Molecular function, Post-translational modification and Technical term. A more complex and widely used vocabulary is that of the Gene Ontology (GO) in which relations between terms have a number of explicit meanings which can be used to make further inferences – such as eukaryotic transcription factors may be located in the nucleus. GO annotations are routinely used for the functional analysis (typically enrichment analysis) of many data types, such as differential expression data. GO provides almost 40,000 terms grouped in three categories: the molecular functions a gene product performs, the biological processes it is involved in and the cellular components it is located in [2]. GO annotations are created manually, by expert curators, as well as by automatic systems such as HAMAP. In addition, annotation projects can also involve students such as the Community Annotation with Ontologies (CACAO). The manual curation of GO terms is a central part of the workflow at UniProt, and UniProt is an active member of the GO consortium. Many UniProtKB keywords are also mapped to equivalent GO terms, and the occurrence of a KW annotation allows the annotation of the equivalent GO term (http://www.ebi.ac.uk/GOA/Keyword2GO).

This publication describes a project initiated by the SwissProt virus annotation team to study the complex interactions of viruses and their hosts and to encode this knowledge in a set of UniProt keywords, GO terms, and interlinked ViralZone pages. All organisms display an impressive battery of innate and acquired antiviral defenses. The viruses we observe today are ‘escape artists’ with a talent for evading these defenses, and whose ability to manipulate their host is essential for virus survival. Their genomes are generally extremely small, encoding few proteins, and these need to be very efficient – they often attack several key points of the host biology. For example, the Hepatitis B virus encodes only four proteins; the polymerase, capsid and surface proteins are essential for the basic life cycle of the virus while the HBX protein plays a major role in host immune defense evasion and cell transformation [3]. Because of the limited coding capacity of most viral genomes, these entities have evolved surgical strikes to cut off multiple essential host defense pathways.

An extensive study of the recent literature was performed to identify essential and conserved host-virus interactions. The number of interactions to consider is significant: as an example, the 13 proteins of HIV-1 have been shown to interact with more than 2589 human proteins [4], although not all such interactions may be physiologically relevant. We have focused on interactions that have been described for at least two different viruses, and confirmed by several independent laboratories. Our investigation resulted in the identification of 54 conserved concepts relating to host-virus interplay, describing the means by which viruses attack host defenses or modulate cellular physiology to facilitate virus replication and propagation. These common interactions were mostly discovered in the past fifty years, and turned out to be poorly described in annotated databases; they are mostly linked to the evasion of conserved acquired or innate host-defenses (in vertebrates). The importance of host-defense processes is underlined by the sheer quantity of viruses counteracting them; one human pathway, the RIG-like receptor (RLR) pathway, is interfered with in some way by at least 14 of the 32 virus families that infect vertebrates.

## Materials and Methods

**Figure 1:**
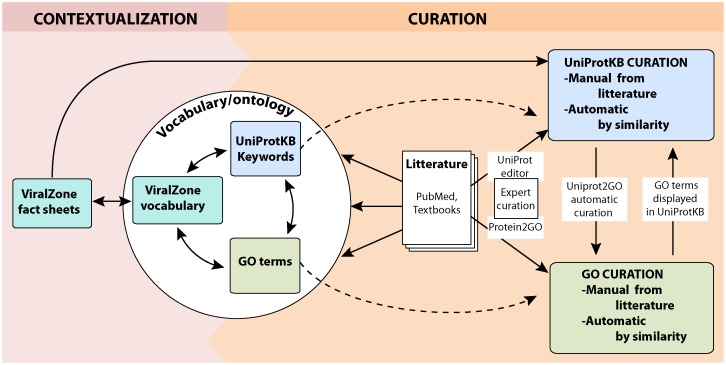
This figure describes the relationship between ViralZone vocabulary, UniProtKB keywords and GO terms. It also explains the process of curation of UniProtKB sequences using the new ontology.

This work describes the creation of a common host-virus interaction vocabulary in ViralZone, UniProtKB and the Gene Ontology (GO), resulting in the creation of 14,390 manual and 908,723 automatic annotations (May 2014). Figure 1 illustrates the interactions between each of these elements, and the way virus sequences were curated using this system.

### Creation of new UniProtKB/SwissProt keywords

At the inception of this project, only two UniProtKB keywords were applicable to protein sequences involved in interaction between virus and host. These were the keyword “Host-virus interaction” – used for any type of interaction between viral and host proteins – and “viral immunoevasion”, used to group viral proteins counteracting the host immune system. In this work we created 63 new keywords to capture all host pathways commonly usurped by viruses (Table 1). To design these terms, we performed an exhaustive review of peer-reviewed literature concerning the interactions between host and viruses, with strong emphasis on viruses affecting humans (interactions between phages and bacteria were not considered in this work). Knowledge from textbooks and reviews was also considered. New keywords were only defined for host pathways affected by several different viruses or molecular mechanisms - keywords were not created for processes affected by a single viral protein. 54 of these new terms described specific interactions between virus and host such as “Inhibition of host RIG-I by virus”. 10 are parent terms, such as “Inhibition of host cell cycle by virus”, which has 5 child terms such as “G0/G1 host cell cycle checkpoint dysregulation by virus”. These concepts need to be refined constantly to keep pace with the latest biological knowledge, and new keywords will be added to the list once sufficient evidence of their importance is available in the literature.

**Table 1:** List of the 65 host-virus interaction processes.

	KW AC	SwissProt	TREMBL	ViralZone AC	GO term	UniProt2GO
**HOST-VIRUS INTERACTION**	KW-0945	5356	550420	VZ-886	GO:0019048	555776
**Host gene expression shutoff by virus**	KW-1190	694	47399	VZ-1582	GO:0039657	48093
Host translation shutoff by virus	KW-1193	229	209	VZ-1579	GO:0039604	438
Host transcription shutoff by virus	KW-1191	316	21221	VZ-1577	GO:0039653	21537
*>Inhibition of host RNA polymerase II by virus*	KW-1104	299	21221	VZ-905	GO:0039523	21520
*>Inhibition of host transcription initiation by virus*	KW-1111	16	0	VZ-904	GO:0039602	16
Host mRNA suppression by virus	KW-1192	240	25969	VZ-1756	GO:0039651	26209
*>Inhibition of host mRNA nuclear export by virus*	KW-1099	59	0	VZ-902	GO:0039522	59
*>Decay of host mRNAs by virus*	KW-1132	58	0	VZ-901	GO:0039595	58
*>Inhibition of host pre-mRNA processing by virus*	KW-1103	130	25969	VZ-903	GO:0039524	26099
**Modulation of host cell apoptosis by virus**	KW-1119	189	15583	VZ-1581	GO:0039526	15772
Activation of host caspases by virus	KW-1073	40	0	VZ-910	GO:0039651	40
Inhibition of host apoptosis by viral BCL2-like protein	KW-1081	30	0	VZ-913		
Inhibition of host apoptosis by viral FLIP-like protein	KW-1082	3	0	VZ-911		
Inhibition of host caspases by virus	KW-1085	22	0	VZ-912	GO:0039650	22
**Modulation of host cell cycle by virus**	KW-1121	296	8147	VZ-1636	GO:0060153	8443
G0/G1 host cell cycle checkpoint dysregulation by virus	KW-1077	21	0	VZ-881	GO:0039646	21
G1/S host cell cycle checkpoint dysregulation by virus	KW-1078	140	0	VZ-880	GO:0039645	140
Host G2/M cell cycle arrest by virus	KW-1079	117	8147	VZ-876	GO:0039592	8264
Inhibition of host mitotic exit by virus	KW-1098	17	0	VZ-877	GO:0039593	17
Modulation of host cell cycle by viral cyclin-like protein	KW-1120	4	0	VZ-879		
**Modulation of host ubiquitin pathway by virus**	KW-1130	140	0		GO:0039648	140
Modulation of host E3 ubiquitin ligases by virus	KW-1123	26	0	VZ-3363	GO:0039649	26
Modulation of host ubiquitin pathway by viral deubiquitinase	KW-1127	83	0	VZ-3364		
Modulation of host ubiquitin pathway by viral E3 ligase	KW-1128	31	0	VZ-3362		
Modulation of host ubiquitin pathway by viral ubl	KW-1129	2	0			
**Viral immunoevasion**	KW-0899	1426	94765		GO:0019049	96191
Modulation of host dendritic cell activity by virus	KW-1118	4	0		GO:0039673	4
Modulation of host NK-cell activity by virus	KW-1131	6	0		GO:0039671	6
Modulation of host immunity by viral IgG Fc receptor-like protein	KW-1124	2	0			
Evasion of host immunity by viral interleukin-like protein	KW-1125	11	0			
**Inhibition of host adaptive immune response by virus**	KW-1080	86	0		GO:0039504	86
Inhibition of host TAP by virus	KW-1107	15	0	VZ-817	GO:0039589	15
Inhibition of host tapasin by virus	KW-1108	7	0	VZ-818	GO:0039591	7
Inhibition of host MHC class I molecule presentation by virus	KW-1115	55	0	VZ-819	GO:0046776	55
Inhibition of host MHC class II molecule presentation by virus	KW-1116	45	0	VZ-820	GO:0039505	45
Inhibition of host proteasome antigen processing by virus	KW-1117	6	0	VZ-815		
Inhibition of host chemokines by virus	KW-1086	12	0	VZ-813	GO:0039553	12
Inhibition of host complement factors by virus	KW-1087	130	29471	VZ-811	GO:0039573	29601
**Activation of host NF-kappa-B by virus**	KW-1074	81	8147	VZ-841	GO:0039652	8228
**Inhibition of host NF-kappa-B by virus**	KW-1100	82	0	VZ-695	GO:0039644	82
***INHIBITION OF HOST INNATE IMMUNE RESPONSE BY VIRUS***	KW-1090	1003	16710		GO:0039503	17713
**Inhibition of host IFN-mediated response initiation by virus**	KW-1113	618	15583	VZ-875	GO:0039502	16201
Inhibition of host TLR pathway by virus		5	0	VZ-3736	GO:0039722	5
Inhibition of host RIG-I by virus	KW-1088	189	0	VZ-856	GO:0039540	189
Inhibition of host MDA5 by virus	KW-1089	28	0	VZ-603	GO:0039554	28
Inhibition of host IRF3 by virus	KW-1092	145	0	VZ-757	GO:0039548	145
Inhibition of host IRF7 by virus	KW-1093	57	0	VZ-653	GO:0039557	57
Inhibition of host MAVS by virus	KW-1097	116	15583	VZ-704	GO:0039545	15699
Inhibition of host TBK1 by virus	KW-1223	5	0	VZ-4477	GO:0039723	5
Inhibition of host IKBKE by virus	KW-1224	28	0	VZ-4478	GO:0039724	28
Inhibition of host TRAFs by virus	KW-1110	79	0	VZ-715	GO:0039547	79
**Inhibition of host interferon signaling pathway by virus**	KW-1114	559	1127	VZ-883	GO:0039502	1686
Inhibition of host interferon receptors by virus	KW-1091	3	0	VZ-843	GO:0039511	3
Inhibition of host IRF9 by virus	KW-1094	43	0	VZ-683	GO:0039560	43
Inhibition of host JAK1 by virus	KW-1096	24	0	VZ-784	GO:0039576	24
Inhibition of host PKR by virus	KW-1102	165	0	VZ-554	GO:0039580	165
Inhibition of host STAT1 by virus	KW-1105	110	1127	VZ-282	GO:0039563	1237
Inhibition of host STAT2 by virus	KW-1106	75	1127	VZ-257	GO:0039564	1202
Inhibition of host TYK2 by virus	KW-1112	35	0	VZ-720	GO:0039574	35
Modulation of host PP1 activity by virus	KW-1126	54	0	VZ-803	GO:0039586	54
**Modulation of host antiviral effects**				VZ-1580		
Inhibition of host tetherin by virus	KW-1084	55	0	VZ-665	GO:0039587	55
Inhibition of host ISG15 by virus	KW-1095	67	0	VZ-723	GO:0039579	67
PML body inhibition by virus				VZ-1676		
Inhibition of APOBEC3G by virus				VZ-3017		
**Suppressor of RNA silencing**	KW-0941	217	31308	VZ-891		
**Activation of host autophagy by virus**	KW-1072	178	798	VZ-846	GO:0039520	976
**Inhibition of host autophagy by virus**	KW-1083	160	0	VZ-845	GO:0039521	160
**Modulation of host chromatin by virus**	KW-1122	63	0	VZ-899	GO:0039525	63
**TOTAL**	**65**	**14607**	**908723**	**57**	**57**	**922941**

KW AC: UniProtKB keyword accession number; SwissProt: the number of times this keyword has been associated with UniProtKB entries by expert curation; TREMBL: the number of times this keyword has been associated with UniProtKB entries by automatic propagation; ViralZone AC: The address code of ViralZone pages corresponding to this keyword. All ViralZone pages can be accessed by using their associated code (XXX) in the address http://viralzone.expasy.org/all_by_species/XXX.html; GO term: the GO term ID linked to the UniProtKB keyword; UniProt2GO: estimated number of GO automatic annotation propagated from UniProtKB keywords through the UniProt2GO pipeline.

### Creation of new ViralZone keyword pages

**Figure 2:**
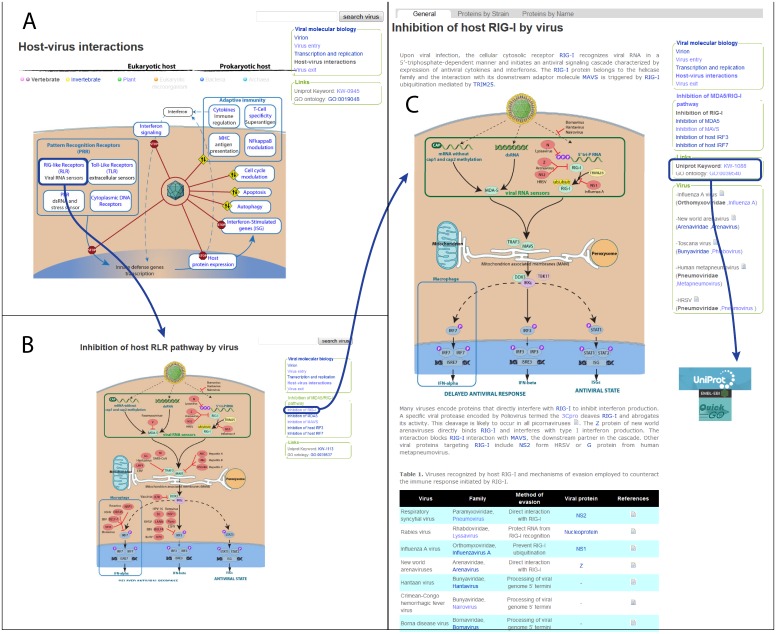
Host virus interactions in ViralZone A) Main entry page (http://viralzone.expasy.org/all_by_species/886.html). All global processes are listed and some links allow browsing down in the controlled vocabulary hierarchy. B) Inhibition of host RLR pathway by virus ViralZone. An illustration describes the RLR antiviral signaling pathway. Known viral proteins inhibiting this pathway are indicated in red circles. A side menu allows reaching description pages for each part of the pathway. (**C**) “Inhibition of host RIG-I by virus” page describes the host process targeted by viruses, and displays viral proteins inhibiting this activity coded by *Lyssavirus*, *Arenavirus*, *Influenzavirus* A and *Pneumovirus*. The text describes this host-virus interaction at a molecular level, and a table displays known viruses and their method used to evade RIG-I antiviral defense. To the left, links to the corresponding UniProtKB keyword and GO term allow users to visit these resources and access to all annotated proteins related to the corresponding ontology term (Figure 4). The field “virus” contains the list of viruses attacking this process, a publication source, and a link to ViralZone virus fact sheet for each virus. Most pages give direct access to reviewed UniProtKB entries annotated with corresponding keyword within “protein by strain” and “protein by name” tabs. An illustration describes the RLR antiviral signaling pathway, and identified viral proteins interfering with RIG-I are indicated in red circles.

**Figure 3:**
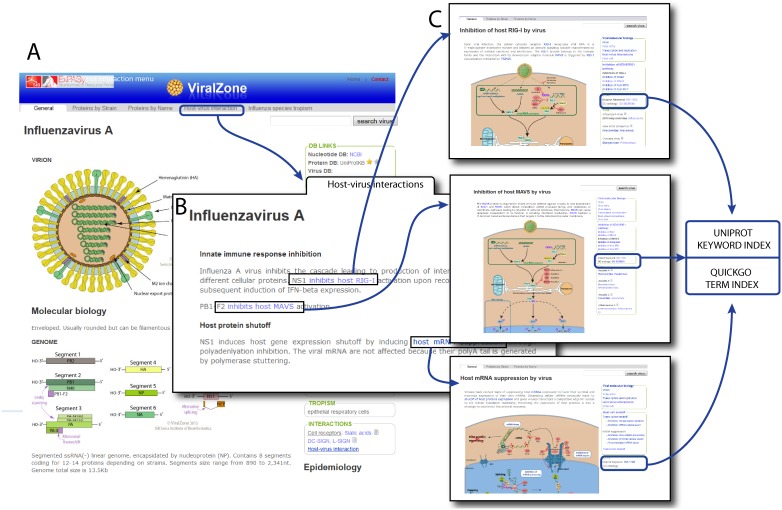
ViralZone fact sheet for *Influenza virus A* (http://viralzone.expasy.org/all_by_species/6.html) contains information about virion, molecular biology and cross references to different sequence databases. A tab “Host-virus interactions” gives access to essential interactions occurring during Influenza A infection. In turn, these interactions are linked to the controlled vocabulary pages where more information concerning the particular interaction of interest is available. From there, users can visit UniProtKB or QuickGO pages related to the keyword or corresponding GO term.

Representation of pathways remains a challenging task in the field of curation, and the relationship between individual proteins which are part of a larger process is often not easy to describe in text form. To facilitate understanding of how viruses impact their hosts we therefore developed 57 ViralZone pages corresponding to the majority of the newly created keywords (Table 1). Each includes a manually created illustration of the host pathway described in the keyword, with points of interaction with viral proteins clearly marked. These ViralZone controlled vocabulary pages also provide a list of all annotated UniProtKB/SwissProt entries containing the keyword as well as all the PubMed references used to generate this annotation. They can be accessed either by host-virus interactions menu (Figure 2) or by fact sheets linking all related process (Figure 3).

### Mapping of UniProtKB/SwissProt keywords to GO ontology

The GO editorial team at EMBL-EBI collaborated with the ViralZone team to update the Gene Ontology (GO) with terms corresponding to the new host-virus interaction ontology. This effort led to the development of 57 GO terms (most newly created) exactly matching new UniProtKB keywords (Table 1). In some cases, one keyword was used to create several GO terms detailing individual aspects of specific functional interactions. The keyword “Inhibition of RIG-I by virus” gave rise to 3 GO terms, specifying different modes of RIG-I suppression - “suppression by virus of host RIG-I via RIG-I binding” and “suppression by virus of host RIG-I activity by viral RNA 5′ processing” – and the functional impact on RIG-I - “suppression by virus of host RIG-I K63-linked ubiquitination”. UniProtKB keywords are not designed to provide such a high level of functional detail.

### Viral gene product curation with the new ontology

**Figure 4:**
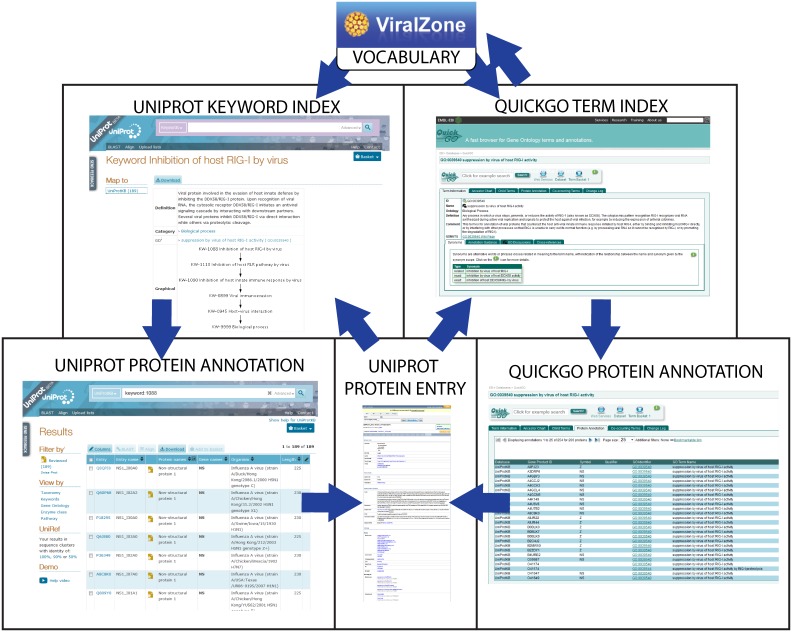
Relationship between ViralZone, UniProtKB and QuickGO databases. Because most host virus-interactions vocabulary or ontology share the same concept, users can browse in the three databases and have easy access to different formats about the same process. Links are indicated by large arrows.

The goal of this work was to curate viral reference proteomes in order to capture host-virus interactions. This curation has been performed in different ways (Figure 1). Keywords were manually curated in UniProtKB/SwissProt records describing viral proteins after careful reading of the literature, using an editor available only to UniProtKB curators. GO terms were also curated manually using the Protein2GO editor, and also annotated automatically (Table 1) based on the mapping to curated keywords. Note that manual of KWs and GO terms is subject to a quality check to ensure the relevance of the information added. The manner in which UniProtKB and QuickGO provide access to information on host-virus interactions is shown in figure 4.

## Results

**Figure 5:**
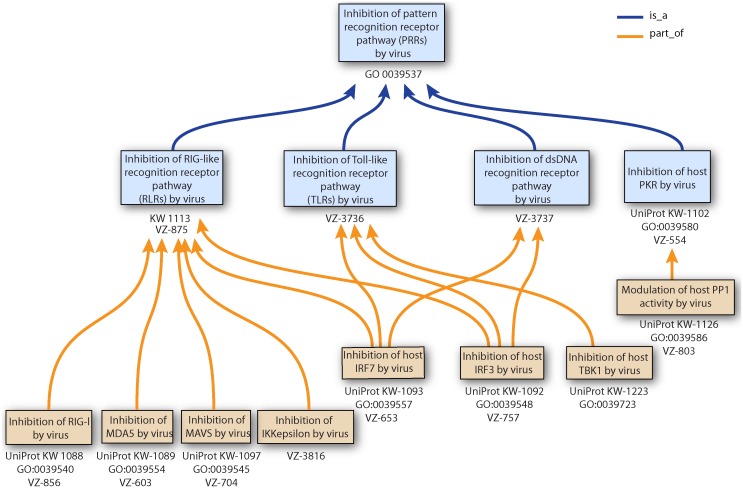
This picture is an example of ontology parent-child relationship. This tree consists of terms used to annotate viral gene products that inhibit the different host components involved in the pattern recognition receptor pathway. Term hierarchy come from ViralZone controlled vocabulary. If equivalence exists, UniProtKB keyword or GO term accession numbers are indicated.

Each organism is the potential target of dozens of viruses [5], which they resist by developing efficient and complex antiviral defenses. Viruses in turn have evolved elaborate mechanisms to escape, neutralize or even exploit these defenses, veritable escape artists that survive in a hostile environment. We have made an extensive study of publications in order to identify the most common modes of interplay between eukaryotic hosts and viruses. We outline some examples of these functional interactions below. The terms and associated annotation are all described in Table 1. An example of the term hierarchy is shown in Figure 5.

### Inhibition of host IFN-mediated response initiation by virus

Upon entering a host cell, viruses must deal with host innate immunity. Pathogen recognition receptors (PRRs) [6], [7] are “foreign” sensors triggered by molecular patterns present in most types of viruses and/or bacteria. Upon activation they induce signaling events that ultimately lead to a cellular antiviral state mediated in vertebrates by the production of interferons and inflammatory cytokines. Many viruses directly block signaling components of these pathways in order to prevent the establishment of the antiviral state. RIGI is a PRR activated by cytoplasmic 5′-triphosphate-RNA [8], a type of RNA that appears in the cell cytoplasm of virus-infected cells. Upon recognition of this ligand, RIGI initiates a cascade resulting in the expression of antiviral genes and interferon beta. This induced antiviral state is potent at preventing virus replication and exit. To replicate in vertebrate cells, many RNA viruses including influenza virus, human metapneumovirus, arenavirus, or poliovirus inhibit RIGI through different strategies [9]. The RIGI downstream effector MAVS is targeted by the hepatitis viruses A [10], B [11] and C [12], suggesting a crucial role for MAVS in repressing these viruses in hepatocytes. Many other viruses counteract downstream key effectors of the pathway through direct interaction with IRF3, IRF7 or NF-kappaB transcription factors (figure 5).

### Inhibition of host interferon signaling pathway by virus

Upon virus pattern recognition, most vertebrate cells release interferon (IFN) alpha or beta. IFNs signal infection to neighboring cells [13]. They bind to the cellular IFNalpha/beta receptors and trigger a signaling cascade that activates hundreds of antiviral genes [14]. Signaling from IFN receptors involves phosphorylation of proteins of the signal transducers and activators of transcription family (STAT). These migrate into the nucleus and activate interferon stimulated genes (ISG). This innate defense pathway is so efficient that almost all vertebrate viruses encode proteins to block it. They disrupt the pathway by degradation or inactivation of different cellular proteins involved: interferon receptors, JAK kinases, STAT1 and 2, or IRF9.

### Modulation of host antiviral effectors

Both PRR and interferon signaling trigger an antiviral state in which more than 300 interferon stimulated genes (ISGs) are up regulated [15]. These gene products display a wide range of activities that together contribute to inhibition of virus replication: messenger and ribosomal RNA degradation, inhibition of cellular translation, viral genome hyper mutation, capsid sequestration, and inhibition of budding. Some ISGs target specific classes of viruses, like APOBEC3G which induces mutations in ssDNA [16] and is efficient against retroviruses. Others are specific for cellular compartments or components like PML proteins, which display an antiviral activity against nuclear replicating viruses [17].

The long history of viral and host coevolution has seen the development of a variety of evasive adaptations to circumvent or inactivate these antiviral effectors. One of the most early and effective means to do so is to prevent ISG transcription and the establishment of the antiviral state. This early attempt at evasion may fail though, and latent viruses will eventually have to face antiviral effectors. Therefore many viruses have evolved other ways to rescue their replication cycle by inactivating key effectors of cellular defense. For example the HIV-1 vif protein counteracts APOBEC3G activity [18], while Herpes simplex virus ICP0 protein prevents PML activity [19].

### Inhibition of host adaptive immune response by virus

In innate immunity, pathogen-specific receptors are encoded by the genome, whereas in adaptive immunity these receptors are “acquired” during the lifetime of the organism. This immunity is said to be “adaptive” because it can memorize the pathogen offence and prepare the body to fight future challenges.

Adaptive immunity relies on the distinction between the bodys self-antigens and the foreign-antigens of unwanted invaders. Infected cells display viral antigens through major histocompatibility complexes (MHCs). These are recognized as non-self by T-lymphocytes, thereby triggering the adaptive response and inducing destruction of the infected cells and the synthesis of neutralizing antibodies. To escape this defense, many viruses inhibit MHC peptide presentation [20]. The herpes virus simplex 1 protein ICP47 binds to the peptide binding site of the transporter associated with antigen (TAP) and inhibits the first step of the translocation pathway [21]. Other strategies for preventing antigen presentation include inhibition of host tapasin, proteasome or class I and II MHC molecules. Viral superantigens are proteins secreted that bridge nonspecifically MHC to T-cell receptors, thereby interfering with the specificity of MHC antigen presentation.

### Modulation of adaptive immunity: inhibition/activation of host NF-kappaB by virus, inhibition of host chemokines by virus

In response to viral infection, the NFkappaB transcription factor can be activated and induce the production of numerous cytokines and chemokines by different cell types including macrophages, dendritic cells or epithelial cells [22]. Some viruses produce their own interleukins, chemokine regulators, or IgG Fc receptor-like proteins [23] which either inhibit the immune response or attract lymphoid cells (in the case of lymphotrophic viruses). The cytomegalovirus gene UL146 encodes for a product similar to cellular alpha chemokine, which attracts neutrophils. These are in turn infected and carry the virus to new sites in the organism [24].

### Suppressor of RNA silencing by plant and insect infecting viruses

In higher plants and insects, post transcription gene silencing (PTGS, also known as RNA interference, RNAi) operates as an adaptive antiviral defense mechanism [25]. Double-stranded RNA molecules from viruses are processed into small single-stranded molecules (short interfering RNA, or siRNA), that hybridize to viral RNAs and target them to the degradation pathway. To counteract this almost all plant viruses and many animal viruses encode (viral) suppressors of RNA silencing (VSRs) which inhibit key steps of PTGS. The enamovirus P0 [26] and cucumovirus suppressor 2b proteins [27] inhibit RNA silencing through inhibition of argonaute 1/AGO1, a component of the RNA silencing pathway. The insect flock house virus B2 protein (FHVB2) suppresses siRNA biogenesis by inhibiting host RISC [28].

### Host gene expression shutoff by virus

After genome replication, and in the late phase of the infection, many viruses synthesize a large number of structural proteins and assemble a huge number of virions within the cell. The high demand for viral protein synthesis is often supported by shutoff of host gene expression, ensuring that all cellular resources are devoted to viral synthesis [29]. Host shutoff prevents also the activation of innate defenses. Host shutoff can be achieved by modulating host transcription, mRNA processing and translation.

Inhibition of host transcription can be mediated by preventing host RNA polymerase II initiation. The TATA-binding protein (TBP) is targeted by the adenovirus E1A protein, which disrupts the interaction between the TBP and the TATA box [30]. The thogoto virus ML protein targets the general transcription factor IIB [31]. RNA polymerase II itself may be directly targeted, for example by the alphavirus nsP2 protein which induces rapid degradation of Rpb1, a catalytic subunit of RNA polII [32].

Host messenger RNAs can be modulated at various stages including pre-RNA processing, nuclear export or modification of RNA decay. Whatever the means, viral messenger RNAs rely on alternative ways to be expressed. For example, viruses shutting off host nuclear gene transcription or mRNA export are often transcribed in the cytoplasm.

The main mechanism by which viruses inhibit host gene expression is by specifically targeting the translation of host mRNAs – which requires proteins not used for translation of viral proteins. Viruses inhibiting cap dependent translation use an internal ribosome entry site (IRES) or similar structures for translation initiation of their proteins [33]. The protease 3C from enteroviruses cleaves host eiF5B [34] while the protease from retroviruses cleaves eiF4G, completely abrogating cellular translation [35].

### Modulation of host cell cycle by virus

Viral replication is limited by the ability of the host metabolic machinery to produce the resources – nucleic acids and proteins – necessary for the assembly of viral progeny [36]. These resources are most abundant during the S-phase of the cell cycle, and many DNA viruses modulate the G1/S transition to initiate DNA synthesis. Viruses such as Epstein-Barr virus, human cytomegalovirus, adenoviruses and SV40 modulate the activity of retinoblastoma (RB) protein family members to drive cells in S-phase [37]–[39]. Viruses infecting quiescent cells have also evolved mechanisms to force entry into the cell cycle. Myxomavirus M-T5 promotes phosphorylation, ubiquitination and degradation of the cyclin-dependent kinase inhibitor p27/KIP1 [40]. Viruses also retard the initiation of mitosis – the G2/M transition – to allow replication of their own genome before mitosis and sometimes to prevent clonal expansion of infected lymphocytes [41].

### Inhibition or activation of host autophagy pathway by virus

Several pathogens interfere with or exploit the host autophagic pathway for their life-cycle or in order to evade or immune responses [42]. Autophagy is a fundamental eukaryotic cellular process for maintaining homeostasis by degrading cellular proteins, organelles and intracellular pathogens. This process is tightly associated with innate and adaptive immunity. The autophagic machinery may promote the production of type-I interferon (IFN) by delivering the cytosolic replication intermediates to the lysosomes and thereby activating endosomal toll-like receptors (TLRs) [43]. Moreover, lysosomal degradation of cytoplasmic compounds contributes to the pool of MHC class II displayed peptides. Herpes simplex virus ICP34.5 interacts with host Beclin-1 and interferes with autophagosome maturation and antigen presentation in dendritic cells [44]. A similar function has been attributed to protein TRS1 from Human cytomegalovirus that modulates Beclin-1. Alternatively, some viruses use autophagy to generate intracellular membranes useful for viral replication. For example autophagy may serve as a generator of intracellular membrane vesicles for picornavirus replication [45].

### Modulation of host cell apoptosis by virus

In order to prevent viruses from spreading within the infected host organism, cells can commit apoptosis, a genetically controlled program of cell death. Many viruses have evolved strategies to inhibit apoptosis by blocking both intrinsic and extrinsic host-initiated cell death pathways. Several adenoviruses, herpesviruses and poxviruses encode proteins that are homologous to the cellular anti-apoptotic Bcl-2 protein. These viral proteins sequester pro-apoptotic Bcl-2 family members including Bak and Bax, thereby inhibiting apoptosis. Viral FLIPs (vFLIPs) block the interaction of the death receptor-adapter complex with the cellular effector FLICE (caspase-8) to prevent the initiation of the downstream caspase cascade.

## Discussion

The host-virus vocabulary presented here consists of 57 ViralZone terms, 65 UniProtKB keywords and 57 corresponding GO terms, describing most of known interactions between viruses and their hosts. The terms provide comprehensive coverage of the mechanism used by the virus families known to infect eukaryotic hosts. While most of current knowledge on host-virus interactions is covered by these terms, our systematic approach will allow expanding and updating the system. Indeed this area of knowledge has grown much in the past ten years, and will presumably continue to develop in the future. Our efforts to create eukaryotic host-virus interaction ontology have led to three levels of implementation: global knowledge and facts in ViralZone pages; viral protein annotation in UniProtKB through keywords; and viral gene and protein annotation through GO terms. At the time of writing the keywords provide a total of 923,113 annotations in UniProtKB while the GO terms provide 922,941 annotations. Together these three implementations provide a global view of viral biology, and a means to annotate knowledge, for a wide user community. Several research institutes and public databases have initiated projects involving the annotation of viral genomes, and we hope that the terms and ontologies presented in this article, which are available from the ViralZone, UniProtKB and GO websites, will help them in these efforts.
